# Prevalence of Overweight and Obesity in Jamaica From 2000 to 2016

**DOI:** 10.7759/cureus.34907

**Published:** 2023-02-13

**Authors:** Kevoyne H Chambers, Rysheme M Reid, Shania C Samuels, Sashana S Cranston, Orbin Barnes, Orlando D Palmer

**Affiliations:** 1 Medicine, Jiangsu University, Zhenjiang, CHN; 2 Medicine, Nanjing Medical University, Nanjing, CHN; 3 Medicine, Jinzhou Medical University, Jinzhou, CHN; 4 Hematology and Oncology, University Hospital of the West Indies, Kingston, JAM

**Keywords:** trends, overweight, obesity, jamaica, caribbean

## Abstract

The prevalence of overweight and obesity in Jamaica has been steadily increasing over the past decade and is now a significant health issue. This paper focuses on the trends in the prevalence of overweight and obesity in Jamaica from 2000 to 2016. Overweight and obesity prevalence in adults increased from 43.8% in 2000 to 55.5% in 2016, from 34.2% in 2000 to 47.4% in 2016in adult males, and from 53.0% in 2000 to 63.6% in 2016 in adult females. In children/adolescents aged 10 to 19 years, the prevalence of obesity has doubled between 2000 and 2016. The data shows that the prevalence of overweight and obesity in children/adolescents increased from 5% in 2000 to 11.4% in 2016, from 4.4% in 2000 to 11.0% in 2016 in boys, and from 5.5% in 2000 to 11.9% in 2016 in girls.

## Introduction

The term overweight is defined as a body mass index (BMI) of over 25 kg/m2, while obesity is defined as a BMI of over 30 kg/m2 [1]. Overweight and obesity have become significant global public health concerns with severe health, psychological, and economic burdens [2]. The prevalence of overweight and obesity has been steadily increasing over the past four decades in both developed and developing countries [3]. According to the World Health Organization (WHO), in 2016, more than 1.9 billion adults were overweight, and 650 million were obese.

Being overweight and obese is associated with numerous health complications. The common ones are inflammation, diabetes mellitus, and cardiovascular disease. However, multiple studies have shown that there is an associated cancer burden in people with excess body weight; the ones with the most evidence are breast cancer, endometrial cancer, esophageal adenocarcinoma, and kidney cancers [4]. There is also a link between asthma and obesity in childhood [5]. Studies have also shown that severely obese people are at high risk for depression due to poor body image [6]. Obesity can also have a severe financial burden on a country’s economy [7]. Based on a study conducted in 2015 the direct cost of diabetes mellitus in Jamaica was between US 567 million and US 765 million dollars [8]. In 2017, Jamaica estimated that cardiovascular diseases and diabetes combined will cost US 77 billion dollars over the next 15 years, this involves treatment costs and the loss of productivity from persons who are affected within those two categories alone [9]. These are all health complications that are associated with being overweight and obese. The prevalence of overweight and obesity has increased substantially and is now a significant health concern for Jamaica. In 2016 24.7%, approximately one in four adults in Jamaica were obese. That same year the country ranked 55 out of 191 countries worldwide based on the percentage of the adult population that was obese and ranked fourth among Caribbean Community and Common Market (CARICOM) member states [10].

This report aims to describe the trends in the prevalence of overweight and obesity in Jamaica from 2000 to 2016. Data from the Pan American Health Organization (PAHO) shows a steady increase in the prevalence of overweight and obesity in Jamaica. Analyzing and highlighting the relevance of this data is vital to help reduce the prevalence of overweight and obesity throughout the next decade. Therefore, this may aid in establishing a solid foundation for better analysis of the root problem in the region so that prevention and treatment strategies may be better implemented.

## Materials and methods

Study design

This was a secondary analysis study of data obtained from the PAHO database [11]. The data available for the prevalence of overweight and obesity from 2000 to 2016 among the Jamaican population was analyzed and summarized for both the adult and children/adolescent cohorts.

Data collection

The data used was collected from the PAHO core indicators database. The PAHO core indicators database provides the latest data on health indicators for 49 countries and territories in the Region of the Americas. The primary sources used to create these health indicators are demographic censuses, national health information systems, population surveys, and data from health facilities [12].

Data analysis

Values were expressed as a percentage of the population. The data obtained was organized and tabulated within Microsoft Excel (Microsoft Corp., Redmond, WA, USA). Further analysis to obtain the p-value and correlation of data was done using the SPSS (IBM Corp., Armonk, NY, USA) statistics tool.

## Results

**Figure 1:**
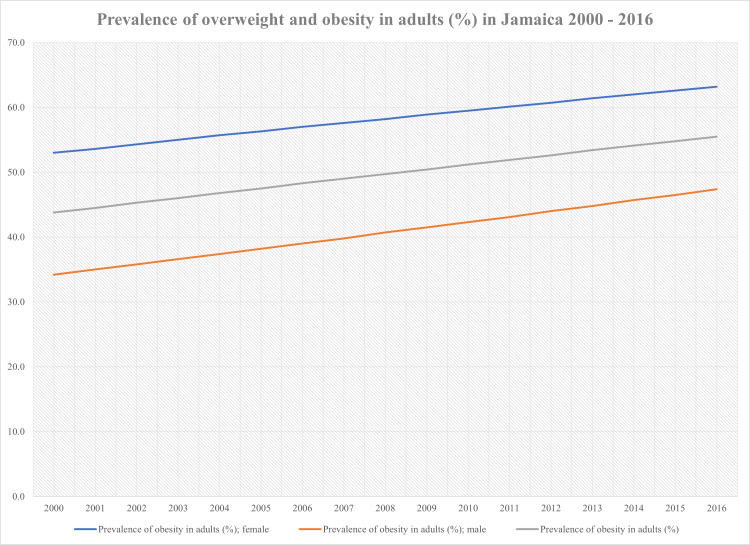
Prevalence of overweight and obesity trends in adults (%) in Jamaica from 2000 to 2016

**Figure 2:**
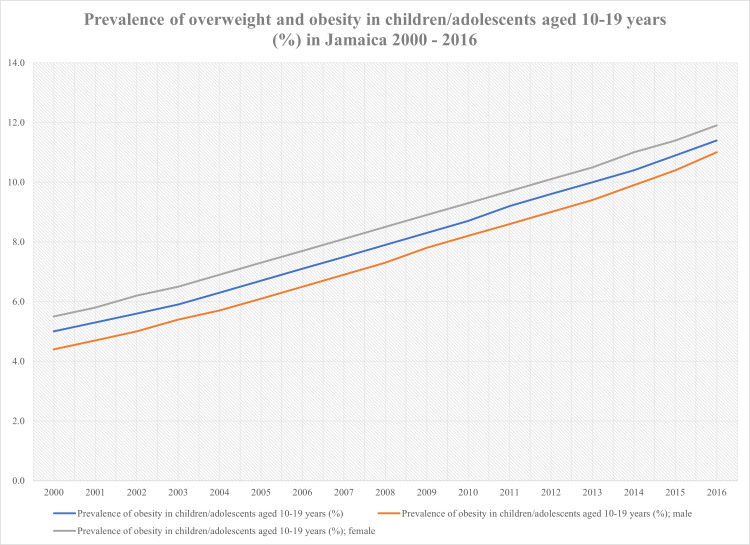
Prevalence of overweight and obesity trends in children/adolescents aged 10 to 19 years (%) in Jamaica from 2000 to 2016

An overview of the data obtained from the PAHO on the prevalence of overweight and obesity from 2000 to 2016 in the Jamaican population is presented in Table 1. Figure 1 highlights the trend of overweight and obesity among adults, while Figure 2 highlights the trend of overweight and obesity among children/adolescents from 2000 to 2016.

**Table 1:** Available data on overweight and obesity prevalence in Jamaica from 2000 to 2016 Source: Pan American Health Organization

Indicator	2000	2001	2002	2003	2004	2005	2006	2007	2008	2009	2010	2011	2012	2013	2014	2015	2016
Prevalence of obesity in adults (%)	43.8	44.5	45.3	46.0	46.8	47.5	48.3	49.0	49.7	50.4	51.2	51.9	52.6	53.4	54.1	54.8	55.5
Prevalence of obesity in adults (%); male	34.2	35.0	35.8	36.6	37.4	38.2	39.0	39.8	40.7	41.5	42.3	43.1	44.0	44.8	45.7	46.5	47.4
Prevalence of obesity in adults (%); female	53.0	53.6	54.3	55.0	55.7	56.3	57.0	57.6	58.2	58.9	59.5	60.1	60.7	61.4	62.0	62.6	63.2
Prevalence of obesity in children/adolescents aged 10-19 years (%)	5.0	5.3	5.6	5.9	6.3	6.7	7.1	7.5	7.9	8.3	8.7	9.2	9.6	10.0	10.4	10.9	11.4
Prevalence of obesity in children/adolescents aged 10-19 years (%); male	4.4	4.7	5.0	5.4	5.7	6.1	6.5	6.9	7.3	7.8	8.2	8.6	9.0	9.4	9.9	10.4	11.0
Prevalence of obesity in children/adolescents aged 10-19 years (%); female	5.5	5.8	6.2	6.5	6.9	7.3	7.7	8.1	8.5	8.9	9.3	9.7	10.1	10.5	11.0	11.4	11.9

The data showed that the prevalence of overweight and obesity among the Jamaican population had been steadily increasing from 2000 to 2016, with the prevalence of overweight and obesity among the children/adolescents population doubling. From 2000 to 2016, there was a 13.2% and 10.2% increase in the prevalence of overweight and obesity in adult males and adult females, respectively. The average prevalence of overweight and obesity over the 16 years for adult males was 40.71% (standard deviation (SD)=4.16), while for adult females it was 49.69% (SD=3.70). In the children/adolescents population, there was a 6.6% increase in the male cohort and a 6.4% increase in the female cohort. The average prevalence of overweight and obesity over the 16 years for boys was 7.43% (SD=2.07), while for girls it was 8.45% (SD=2.02). Overall, the increase in the prevalence of overweight and obesity in the adult population was 10.2% with an average of 58.12% (SD=3.23) and 6.4% in the children/adolescents population with an average of 7.99% (SD=2.04).

## Discussion

Being overweight and obese are serious and important issues for Jamaica. Obesity-related comorbidities such as diabetes mellitus and cardiovascular diseases have become one of the leading causes of death in the Jamaican population, with cardiovascular diseases alone accounting for 27% of deaths under 70 years [13]. This indicates that appropriate prevention and treatment strategies are both crucial issues for the country. Adolescents/children with obesity are more likely to develop other serious health issues. Childhood obesity is associated with a higher chance of more aggressive asthma attacks, premature death, and disability in adulthood. Additionally, obese children may experience psychological issues such as depression [6,14]. Given the long-term impact of obesity on children and the rapid rate of increase in its prevalence, acknowledging and tackling this issue is essential for the sustained health of Jamaican youth.

In 2000, the prevalence of overweight and obesity was 34.2% in adult males, and 53.0% in adult females. In 2016 these percentages rose to 47.4% in adult males and 63.2% in adult females, which shows that the prevalence of overweight and obesity is much higher in adult females compared to adult males. Another crucial point to consider is that while the prevalence of obesity and overweight is higher in adult females, the absolute increase in adult males was 13.2% (p<0.01). In comparison, it was 10.2% in adult females (p<0.01), suggesting that the prevalence of overweight and obesity is increasing faster in adult males compared to adult females. In 2016, the prevalence for both genders was 55.5%, an 11.7% increase from the prevalence for both genders in 2000, which was 43.8%. For some context, the global prevalence of overweight and obesity in 2016 was 39.0%, which would put the prevalence of overweight and obesity among adults in Jamaica at 16.5% higher than the global average.

In 2000, the prevalence of overweight and obesity in adolescents aged 10 to 19 years was 4.4% in males and 5.5% in females. These figures climbed to 11.0% and 11.9% in 2016. Females consistently maintained a higher prevalence of overweight and obesity throughout the 16 years. However, the absolute increase in the prevalence amongst males in this cohort is a significant observation to note as well. There was a 6.6% increase in males (p<0.01); in comparison, females had a lower absolute increase of 6.4% (p<0.01), indicating that even though the prevalence of obesity and overweight was higher in females, the rate of increase was slightly higher in males. In 2016 the prevalence for both genders was 11.4%, a 6.4% increase from the prevalence for both genders in 2000 which was 5.0%. The global prevalence of overweight and obesity among adolescents in 2016 was 18% [1]. Jamaica’s prevalence of overweight and obesity was only 6.6% lower than the global average.

In 2011 the ministry of health, per the ministry of education in Jamaica, conducted a health-promoting school survey [15]. The objective of this study was to gather data to aid in establishing policies and programs for school health. The study looked at various indicators that affect students' health in 60 schools across the 14 parishes of the country. This was an excellent initiative to promote proper health awareness among children/adolescents. However, this strategy should also be implemented in other sectors of the country. Health education should be provided to all people at all levels on the critical impacts of overweight and obesity on children and adults, thus increasing the public's awareness. This may be achieved through multilevel community and school-based interventions [16]. Additionally, the prevalence of obesity may also be managed by implementing fitness programs in the curriculum of educational facilities and workplaces.

A major limitation of this study is that the data analyzed and presented could only show the prevalence of overweight and obesity in the Jamaican population from a general perspective [17]. The data available do not allow for analysis of the prevalence of overweight and obesity in various subgroups and geographical locations across the country.

## Conclusions

The data reflects a significant increase in the prevalence of overweight and obesity in adults and children/adolescents in the Jamaican population between 2000 and 2016. For both age groups, females consistently had a higher prevalence than males. However, the data for males in both cohorts reflected a more significant rate of increase in the prevalence of overweight and obesity between 2000 and 2016.
